# 2-D sex images elicit mate copying in fruit flies

**DOI:** 10.1038/s41598-022-26252-5

**Published:** 2022-12-22

**Authors:** Sabine Nöbel, Magdalena Monier, David Villa, Étienne Danchin, Guillaume Isabel

**Affiliations:** 1 Université Toulouse 1 Capitole and Institute for Advanced Study in Toulouse (IAST), Esplanade de l’Université, 31080 Toulouse Cedex 06, France; 2 Laboratoire Évolution & Diversité Biologique (EDB UMR 5174), Université de Toulouse Midi-Pyrénées, CNRS, IRD, UPS, 118 Route de Narbonne, 31062 Toulouse, France; 3 Centre de Biologie Intégrative (CBI), CNRS UMR 5169, Université de Toulouse Midi-Pyrénées, Toulouse, France; 4 Centre de Recherches sur la Cognition Animale (CRCA), Centre de Biologie Intégrative (CBI), CNRS UMR 5169, Université de Toulouse Midi-Pyrénées, Toulouse, France; 5 Department of Zoology, Animal Ecology, Martin-Luther University Halle-Wittenberg, Hoher Weg 8, 06120 Halle (Saale), Germany

**Keywords:** Ecology, Behavioural ecology, Evolution, Cultural evolution, Sexual selection

## Abstract

Although the environment is three-dimensional (3-D), humans are able to extract subtle information from two-dimensional (2-D) images, particularly in the domain of sex. However, whether animals with simpler nervous systems are capable of such information extraction remains to be demonstrated, as this ability would suggest a functional generalisation capacity. Here, we performed mate-copying experiments in *Drosophila melanogaster* using 2-D artificial stimuli. Mate copying occurs when naïve females observe the mating success of potential mates and use that social information to build their own mating preference. By replacing live demonstrations with (*i*) photos or (*ii*) simplified images of copulating pairs, we found that even crudely simplified images of sexual intercourse still elicit mate copying, suggesting that *Drosophila* is able to extract sex-related information even from a degraded image. This new method constitutes a powerful tool to further investigate mate copying in that species and sexual preferences in general.

## Introduction

The high sensitivity of humans to two-dimensional (2-D) images, and particularly so in the context of sex, raises the question of whether that capacity is unique to our species. However, little is known on the perception of flat images in non-human animals^1^, although several zoos empirically began using images to study mate choice in captive animals with the goal of stimulating reproduction by mimicking situations of mate choice^2,3^. More generally, the use of artificial visual stimuli in a wide variety of behavioural experiments is becoming a powerful methodology across all taxa involving clay models, robots, pictures, video playbacks, computer animated stimuli or even the use of virtual reality techniques^3–14^.

Here, we report on a study in the model organism *Drosophila melanogaster*, where artificial visual stimuli have been used for decades mostly in simple forms to study navigation^15,16^. However, all these approaches use the capacity of fruit flies to have a 3-D perception of their environment^17^. As we wanted to investigate whether they also use 2-D images as sources of information and whether they can extract information from them, we modified the protocol to study social learning in the context of mate choice, a form that usually is called mate copying. Mate copying occurs when, after observing another females’ mate choice, an observer female tends to preferentially mate with the same male (“individual based” mate copying) or with males of the same phenotype (“trait based” mate copying) as the one chosen during the demonstration^18,19^.

Mate-copying experiments in the fruit fly consist of a demonstration where a virgin, naïve observer female can watch another female, the demonstrator, copulating with a male of a certain phenotype and a male of a contrasting phenotype being rejected, followed by a mate-choice test where the observer female can mate with one of the two male phenotypes^20^. We showed previously the sophistication of this social-learning strategy in *D. melanogaster* with experiments using demonstrations with real (living) conspecifics^20–26^.

**Figure 1:**
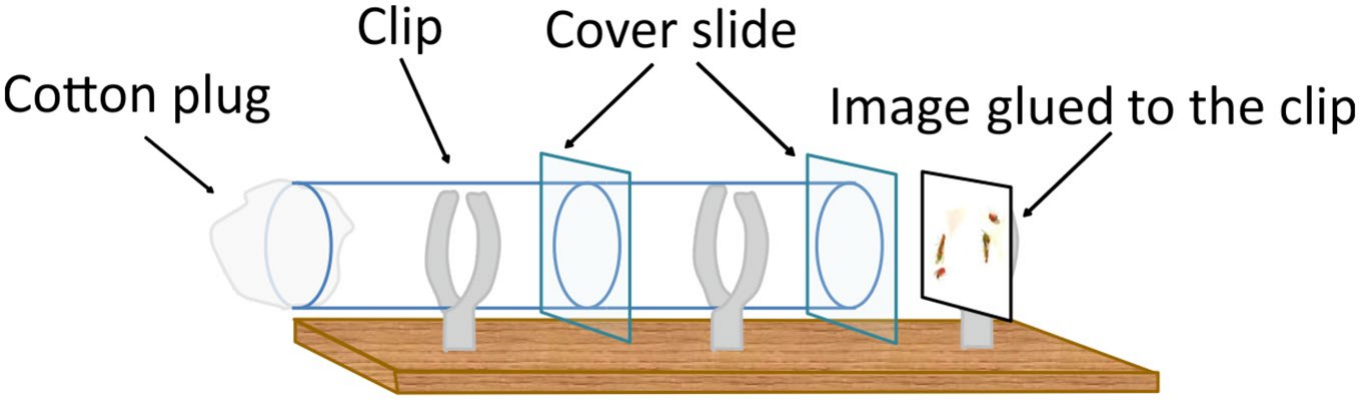
Device used in the mate-copying experiment using images during the demonstration.

**Figure 2:**
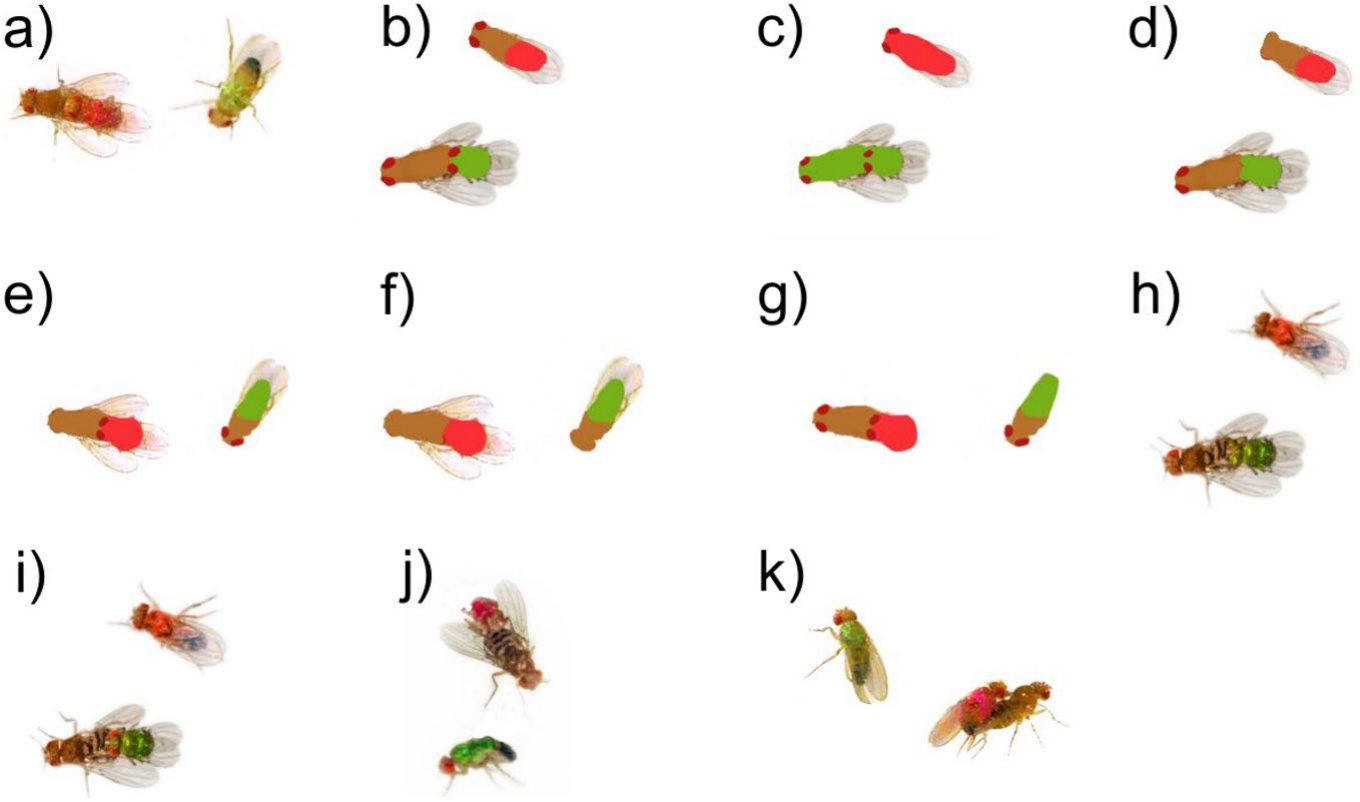
Examples of images used during the demonstration in the various experiments. Here we present only one example per type of treatment, but every treatment included a mixture of top and lateral views of the copulating pairs. (**a**) Unmodified photo where only the background was removed and the colours were made brighter; (**b**) a "dots" demonstration, *i.e.* pink or green area (or dot) on the back of the males; (**c**) a "painted" demonstration, *i.e*., the whole male and whole couple were coloured in pink or green; (**d**) dots but in which males eyes were removed; (**e**) dots in which female eyes were removed; (**f**) dots in which we removed the eyes of all flies; (**g**) dots in which we removed the wings of all flies; (**h**) photo in which we removed the eyes of males; (**i**) photo in which we removed the eyes of the female; (**j**) photo in which we removed the wings of males; (**k**) photo in which we removed the wings of females.

To study whether fruit flies can extract the same kind of information from 2-D images, we thus simply replaced live demonstrations with flat images of a demonstrator female copulating with one male of a given colour, while a male of the other colour was standing by apparently rejected (Fig. 1). The modification of such pictures and images (Fig. 2) further allowed us to identify the important cues for mate copying in fruit flies.

We found that demonstrations with photos or simplified static images in fruit fly mate-copying experiments still elicit as efficient mate copying as live demonstrations, provided that specific traits in demonstrator images, such as wings and eyes, are maintained. Hence, 2-D images of copulating conspecifics do evoke sexual intercourse to a fly, and females perceive artificial demonstrations as actual sexual intercourse to the point that they extract information from them (here the colours of the successful and rejected males). Thus, mate-copying experiments using flat images of sexual intercourse can constitute a powerful tool to further investigate mate copying in that species.

## Results

**Figure 3:**
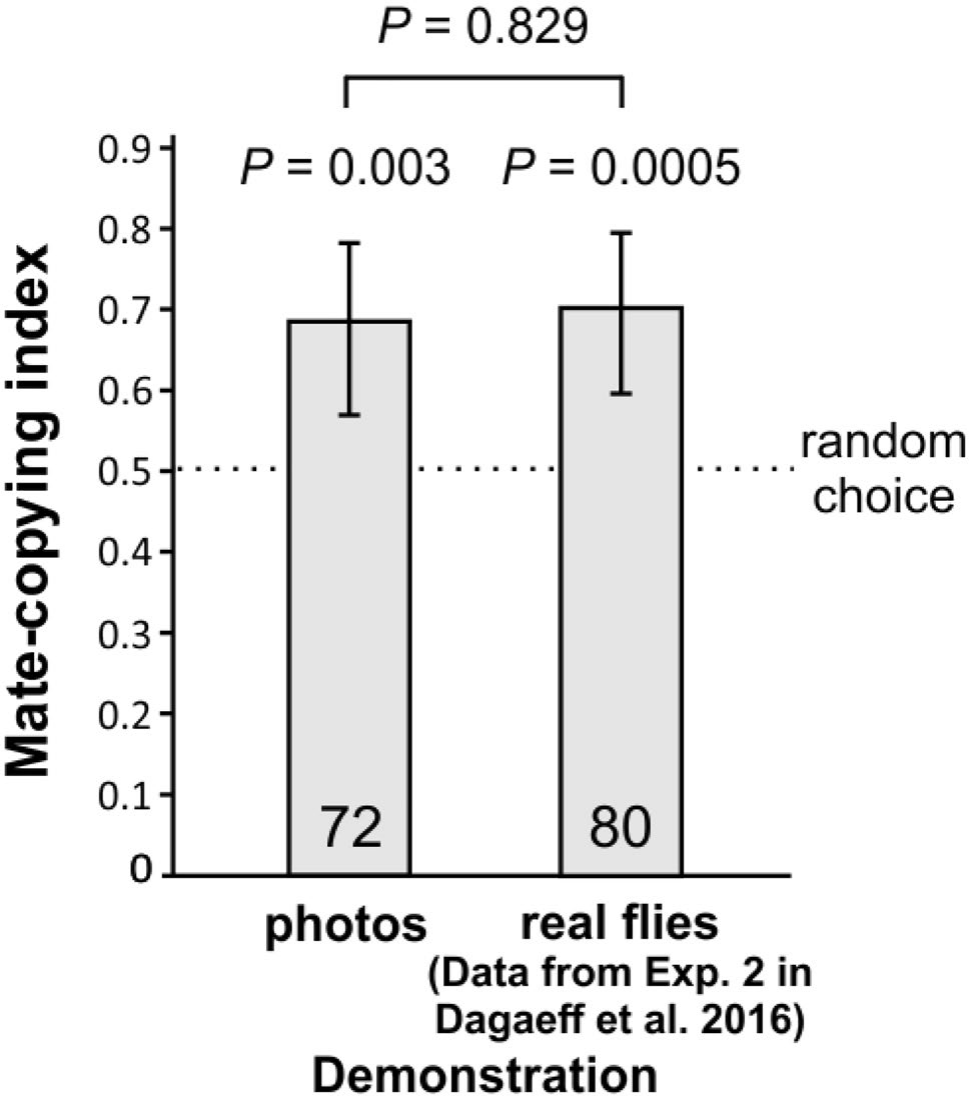
Mate-copying indexes with live or photo demonstrations. Number inside bars: number of trials. Statistics: above bars, *P* values of the binomial tests of departure from random choice (represented by the dashed line); the *P* value above the horizontal bar is that of the comparison between live and picture demonstrations (GLMM). Error bars represent Agresti-Coull 95% confidence intervals. For an example of the photos used see Fig. 2a.

***Experiment 1*** explores the role of visual cues in mate copying by comparing live demonstrations with demonstrations using static photos of a female copulating with one male phenotype plus a rejected male of the other phenotype standing by (for 30 min in both cases). When later given the choice between different green and pink males, observer females showed a significant bias for males of the phenotype chosen by the demonstrator female both after a live demonstration (as previously shown^20^) or after a photo demonstration (binomial test; live demonstrations: *n* = 80, *P* = 0.0005; photo demonstrations: *n* = 72*, P* = 0.003; Fig. 3; effect of copulating male’s colour, Fisher test: *P* = 0.460). Furthermore, there was no significant difference in mate-copying scores between photo and live demonstrations from a previous study^20^ using live demonstrations (GLMM: *n* = 152, *P* = 0.829; Fig. 3). Thus, photos sufficiently evoke sex to elicit efficient social learning about sexual preferences, which shows that mate copying in fruit flies can be efficient with purely visual cues, in the absence of any olfactory cues.

Having shown that females generally copy the information in the images, we next wanted to know the minimum observation time required to trigger mate copying, since copulations typically last 20–25 min^27^.

**Figure 4:**
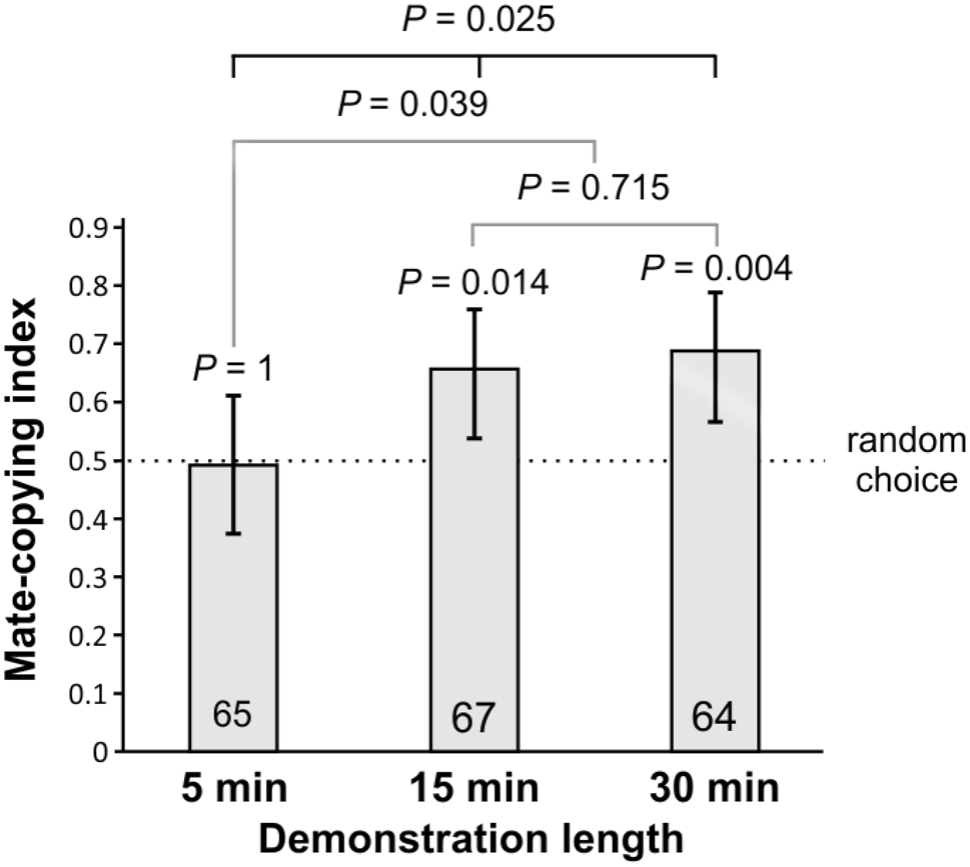
Mate copying with varying demonstration length. Number inside bars: number of trials. Statistics above each bar are the P-values of the binomial tests of departure from random choice (represented by the dashed line), and the one above the horizontal black bar is that of a GLMM comparing the effect of treatment in the three groups. The grey horizontal bars provide the *P* values of a Fisher test to compare the 15 min and 30 min treatments and these two with the 5 min treatment. We used Holms correction for multiple testing. Error bars represent Agresti-Coull 95% confidence intervals.

In*** Experiment 2***, we slightly modified Experiment 1 and varied the duration of the demonstration. In some trials the duration of the photo-demonstration lasted 5 min, in other it lasted 15 min, and in a third treatment it lasted 30 min. Photo-demonstration length significantly affected mate-copying scores (GLMM: *n* = 196, *P* = 0.020, Fig. 4), while previously identified potential confounding factors like photo ID (*P* = 0.993), experimenter ID (*P* = 0.510) or normalized air pressure (*P* = 0.103) were non-significant. 5-min demonstrations did not elicit mate copying (binomial test: *n* = 65, *P* = 1; Fig. 4), while 15-min and 30-min demonstrations elicited significant mate copying (binomial tests: *n* = 67, *P* = 0.014, and *n* = 64, *P* = 0.004, respectively) and did not differ significantly between each other (Fisher test: *n* = 131, *P* = 0.715). These two treatments pooled together differed significantly from the 5 min treatment (Fisher test: *n* = 196, *P* = 0.039).

Next, we took advantage of the fact that photos are easy to manipulate to determine some clues that might be needed for mate copying. Then we dissected the cues that are used by observer females to recognize a sexual intercourse by modifying step-by-step specific characteristics of the pictures used for demonstration.

**Figure 5:**
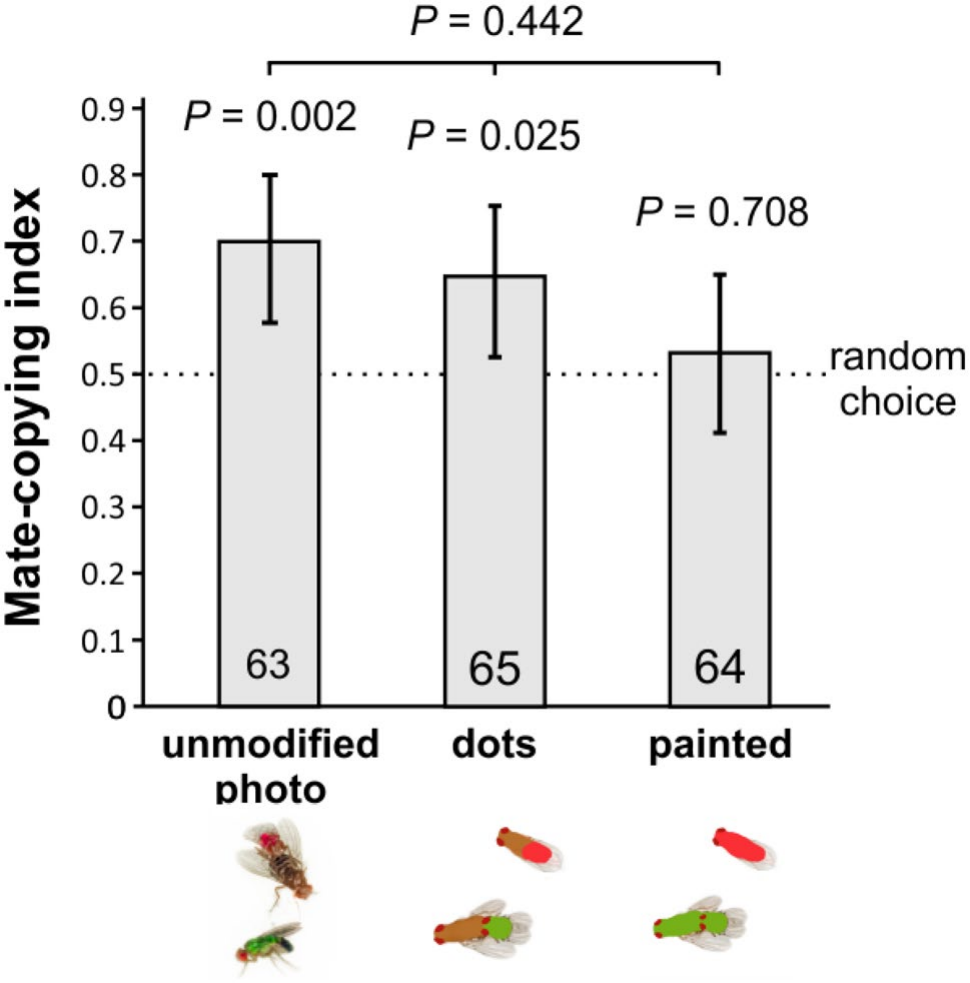
Effect of modification of colour patterns on mate copying. All treatments include a mixture of lateral or top views of the copulating pair. Statistics above bars: *P* values of the binomial tests of departure from random choice (represented by the dashed line); above the horizontal bars: *P* values of binomial tests of a GLMM testing the treatment effect within each panel. Error bars: Agresti-Coull 95% confidence intervals. For examples of the pictures used see Fig. 2a,b,c.

***Experiment 3*** tested whether simplifications of the colour patterns affected on mate copying. There were three treatments (Fig. 5): (1) A control using unmodified photos (replicating Experiment 1, for an example of the pictures used see Fig. 2a); (2) a treatment where male abdomens were replaced with a uniform green or pink patch, and the entire female body (except the eyes) with a brownish uniform patch (thereafter called ''dots'', for an example of the pictures used see Fig. 2b); and (3) a treatment where the whole body of all flies (but not eyes) was replaced with a uniform colour ellipsoid hiding large anatomical details (thereafter called "painted", for an example see Fig. 2c). Again, control observer females performed mate copying (binomial test: *n* = 63, *P* = 0.002; Fig. 5), thus replicating Experiment 1 for a third time. More surprisingly dots demonstrations also elicited significant mate copying (binomial test: *n* = 65, *P* = 0.025), but not painted demonstrations (binomial test: *n* = 64, *P* = 0.708). However, the three-level treatment effect was non-significant [GLMM: *n* = 192, *P* = 0.442, or as a continuous effect (GLMM: *n* = 347, *P* = 0.146); Fig. 5] in an analysis that included the photo ID (*P* = 0.044) plus the normalized air pressure (*P* = 0.09) effects.

Thus, our crude simplification did not eradicate mate copying when it involved only the abdomen but did so when it involved the whole male body, suggesting that pictures of the former treatment but not of the latter, still sufficiently evoked a sexual intercourse to elicit mate copying.

We then went a bit deeper in dissecting the cues that lead observer females to recognize a sexual intercourse by simplifying the demonstration images, first starting from the dot treatment (Experiment 4) or an unmodified picture (Experiment 5). In both cases, we removed either the eyes or the wings.

**Figure 6:**
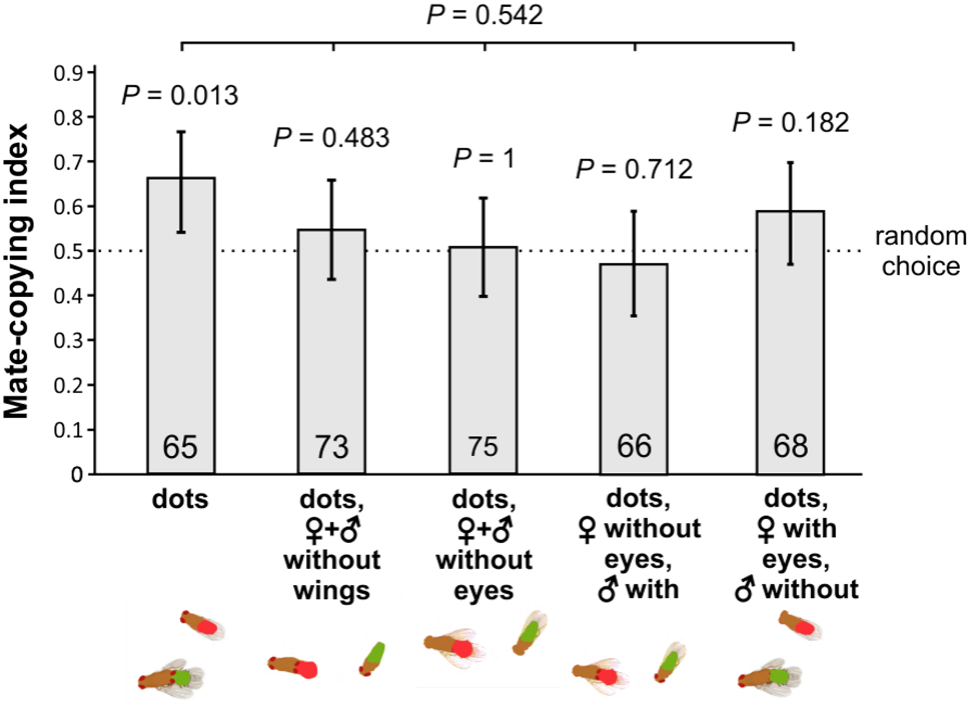
Effect of modifications on the dots treatment on mate copying. All treatment include a mixture of lateral or top views of the copulating pair. Statistics above bars: *P* values of the binomial tests of departure from random choice (represented by the dashed line); above the horizontal bars: *P* values of binomial tests of a GLMM testing the treatment effect within each panel. Error bars: Agresti-Coull 95% confidence intervals. For examples of the pictures used see Fig. 2b,d,e,f,g.

***Experiment 4*** explores the effect of the removal of the eyes or the wings on the capacity of the pictures to evoke sexual intercourse to observer females as revealed by significant mate copying. To test the observer's generalization ability from the degraded images during the demonstration phase to the live flies during the test phase, all modifications were added to the dot treatment (used as a control; for examples of the pictures used see Fig. 2b, d-g). Again, the dots treatment elicited mate copying (binomial test: *n* = 65, *P* = 0.013), but we found that dots without wings, or eyes eradicated mate copying (wings, binomial test: *n* = 75, *P* = 1; eyes, binomial test: *n* = 73, *P* = 0.483, second and third bar of Fig. 6). This suggests that wings or eyes are important cues. Furthermore, keeping only the eyes of males or females from a dot-type of image did not restore mate copying (two right bars of Fig. 6: female eyes missing, binomial test: *n* = 68, *P* = 0.182; male eyes missing, binomial test: *n* = 66, *P* = 0.712).

None of the potential confounding effects [photo ID (*P* = 0.77), experimenter ID (*P* = 0.624) or normalized air pressure (*P* = 0.922)] were significant (Fig. 6). However, a GLMM analysis did not reveal any significant difference among the five treatments (GLMM: *n* = 347, *P* = 0.542). Thus, when using an already simplified picture in the form of the dots-treatment, both eyes and wings seem to be necessary for observer females to copy the simplified versions of the dots treatment.

**Figure 7:**
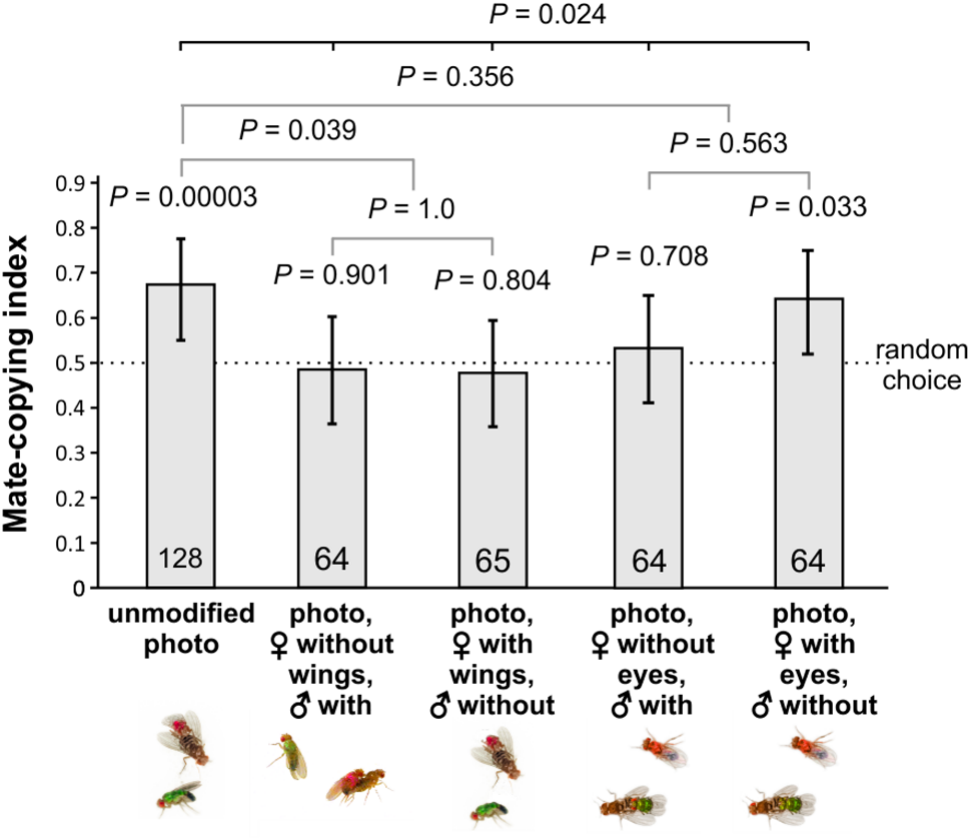
Effect of modifications on the initial photo on mate copying. All treatments include a mixture of lateral or top views of the copulating pair. Statistics above bars: *P* values of the binomial tests of departure from random choice (represented by the dashed line); above the horizontal bars: *P* values of binomial tests of a GLMM testing the treatment effect within each panel. The grey horizontal bars provide the *P* values of a Fisher test to compare treatments pairwise. We used Holm’s correction for multiple testing. Error bars: Agresti-Coull 95% confidence intervals. For examples of the pictures used see Fig. 2a,h,i,j,k.

Since generalizability seems to be very sensitive to the brownish, uniform body of the fly (dots treatments), we conducted*** Experiment 5*** that is similar to Experiment 4 in that it uses the same simplifications, but this time not on the basis of the “dots” treatment but based on unmodified photographs (Fig. 2h,i,j,k). Again, control observer females that saw demonstrations with unmodified photos mate copied (binomial test: *n* = 128, *P* = 0.00003, Fig. 7). Wing removal on females (binomial test: *n* = 65, *P* = 0.804) or males (binomial test: *n* = 64, *P* = 0.908) alone eradicated mate copying (Fig. 7). Contrastingly, although mate copying disappeared when only males had eyes (binomial test: *n* = 64, *P* = 0.708), it remained significant when demonstrator females (but not males) had eyes (binomial test*: n* = 64, *P* = 0.003). Furthermore, the five-level treatment effect was significant (GLMM: *n* = 385, *P* = 0.024), while potentially confounding effects were not (photo ID: *P* = 0.723; normalized air pressure: *P* = 0.333). The treatments manipulating the eyes do not differ from each other (Fisher test: *n* = 128, *P* = 0.563), same for the treatments manipulating the wings (Fisher test: *n* = 129, *P* = 1). A pairwise comparison of the treatments manipulating the eyes vs normal pictures was not significant (Fisher test: *n* = 257, *P* = 0.356), but there was a significant difference between the treatments manipulating the wings vs. normal pictures (Fisher test: *n* = 256, *P* = 0.039). Thus, wings in demonstrator males and females seem to be important cues, while eyes are not. The dissymmetry in the role of female and male eyes is surprising and warrants further investigation.

## Discussion

In this study, we used mate copying as a sensitive biological test to study the visual and integrative capacities of fruit flies in the two-dimensional domain in a series of lab experiments. We decided to use static images of copulating flies as stimuli because screens can be problematic in some cases when they do not fit the requirements of the species in terms of colour vision, brightness, flickering rate etc. (reviewed in^28^) and as copulating flies usually do not move. Furthermore, we know from past studies that only watching the copulating pair plus the rejected male is as efficient at eliciting mate copying as watching the courtship plus copulation^20^. Additionally, we recently showed that observer females copy only the acceptance, but not the rejection of a male^29^. Although courtship and mate choice in *Drosophila* is usually multimodal^30–32^, overall, our experiments show that mate copying in fruit flies can function with purely visual cues only, as photos provide no other information. This suggests that *Drosophila* vision is far more acute than typically assumed. The general view is that fruit flies have poor close-range vision^33,34^ and recognize their conspecifics primarily, if not exclusively, from pheromonal cues^35,36^ and males' courtship songs^37,38^. However, our experiments show that this is only partially true. Even though fruit flies perceive the world in 3-D^17^, surprisingly enough, we found that they also perceive information from static 2-D images, and are able to extract information from them. This indicates that pictures of sexual intercourses are recognised as such, leading females to react to the picture in the same way as they would to a real copulation, as we found. Moreover, they can extract information from those 2-D images, in our case to artificial male coloration, and build a sexual preference accordingly. This is even more surprising that photos specifically fit the human eye, implying that they may not match the requirements for insect vision.

***On a practical point of view***, using image demonstrations in mate-copying experiments can reduce the number of animals involved in experiments, while standardizing and controlling stimuli, as well as simplifying experimental designs and accelerating data acquisition. It also allows the non-invasive manipulation of specific stimuli^39^, while meeting the ethical requirements of the “3Rs” replace, reduce, and refine^40,41^. It also makes it possible to imagine unrealistic stimuli (for example by manipulating the size) to provide virtual information of any kind while avoiding invasive techniques or surgery. Although our result show that vision is sufficient to elicit full mate copying, and in addition to the colours, this does not rule out a potential role of smell or sound. This could be tackled using deaf or anosmic observer females. Furthermore, using images to investigate fruit fly social behaviour would allow to tackle various questions regarding important stimuli^42^, the impact of novel phenotypes^43^, or varying group composition or behaviour^44,45^ and to study social learning in general. In addition, this will accelerate the study of the mechanisms of mate choice in *Drosophila* in ways similar to those performed in humans where images, especially faces, are often used to study attractiveness^41,46–48^. All these characteristics are central to the study of the neurogenetics of mate choice in *Drosophila*.

***On a conceptual point of view***, the fact that photo demonstrations trigger mate copying implies that observer females do interpret them as sexual intercourses and extract social information about male mating success from them. We also found that the preference for an artificially coloured phenotype can similarly be elicited with crudely simplified images of copulating conspecifics if some key characteristics such as eyes and wings are kept. We know that these flamboyant phenotypes do not appear in nature. Thus, our experiments are not providing any information about mating preferences in the wild but reveal surprising cognitive capacities as the importance of the 2-D vision to grasp information, here concerning sex, and provide some details about the relevant cues. To our knowledge, only one study showed that wings are crucial visual stimuli in fly communication in a predator context^49^. In our case, it might be possible that in demonstrations with wingless males, females are not able to generalize the wingless males of the demonstration to the winged real males offered to them during the test, strengthening the suggested importance of the wing presence. The importance of eyes recalls the fact that in humans the presence of patches reminiscent of eyes are central to making a vague image evoke a face (face pareidolia^50^). Furthermore, our results suggest that fruit flies not only recognize conspecifics on the images, but also that these images are sufficiently sexually motivating to stimulate social learning of mate preferences. In the same vein, it has been shown that male flies can initiate courtship towards robotic flies, provided that those robots have roughly the size of a female fly and move at the speed of a fly^51^. Our experiments reinforce the idea that fly brains are “tuned” to recognizing anything that has roughly the size and shape of a fly as a fly, and everything that has roughly the size and shape of a copulating pair as a copulating pair.

## Materials and methods

### Fly maintenance

We used the common laboratory Canton-S strain of *D. melanogaster* for the photos as well as for the behavioural experiments. Flies were raised in 30 ml vials containing 8 ml corn flour-agar-yeast medium at 25.4 ± 0.9 °C and 57 ± 3.6% humidity with a 12:12 h light:dark cycle.

Flies were sexed and sorted without anaesthesia by gentle aspiration within 6 h after emergence and kept in unisex groups of 7 females or 15 males per vial before experiments. Experimental flies were virgin and 3 to 5 days old. Experiments were conducted under the same conditions as breeding. Males and females were used only once. Fly manipulations were performed by gentle aspiration without anaesthesia. After the experiments, observers and demonstrators were euthanized in a freezer (12 h at − 20 °C).

### Images

To take photos, males were dusted in green (Shannon Luminous Materials, Inc. #B-731) or pink powders (BioQuip Products, Inc. #1162R) and got 20–30 min time to clean and rest. Then, a virgin female and one male of each colour were semi-constrained in a square, transparent plastic box 1.8 cm × 1.8 cm, closed with a white foam plug, so that flies could have a volume of about 1 × 1 × 0.4 cm^3^ in which they could walk and interact for several minutes. Photos were taken when one male was mounting the female for several minutes, thus, very likely during insemination.

Photos were taken with a camera Panasonic DMC FZ300 (25–600 mm lens) under white light at 3–5 cm distance to the flies. Photos were then edited with Corel Photopaint to intensify the green and pink dusting of the males and remove the background. On each photo, a couple plus a rejected male of the opposite colour were presented on white background either in top or front view. Angles and position of flies varied between pictures. The size of the flies on the printed photo (printed on photo paper) was about 2.5 mm, which corresponds to their natural size.

Depending on the experiment we did further modifications of the photos as described below.

### Behavioural test

Behavioural tests were performed in a similar set-up as the original speed learning protocol developed by Dagaeff et al.^20^ with slight modifications (Fig. 1). We used the same device with double plastic tubes (1.1 cm × 3 cm each) separated by a microscopy cover slide (1.6 cm × 1.6 cm) but only one side was closed with a cotton plug while a transparent cover slide closed the other side (fixed with Uhu Patafix). The image was placed at 0.9–1.2 cm distance to this cover slide.

First, the observer female was placed in the compartment next to the image, which was covered with white cardboard at that time. For the time of the demonstration the cardboard was removed, and the observer female could watch the image. Later, the image was covered again, and two virgin males dusted in green and pink were introduced for the mate-choice test. The mate-choice test started as soon as the partition was removed, and observer females had 30 min to mate with one of the two males. We recorded the phenotype of the first and second courting male (*i.e*., showing wing vibrations), as well as the phenotype of the male she chose to copulate with.

All replicates were run as blocks of six trials with cardboard separations between experimental set-ups to prevent information exchange between the flies and prevent disturbance by the surrounding. In each block of six trials, three photos showed green males copulating, while the three others showed pink males copulating. The attribution of a photo to the observer fly was random and kept as blind as possible: each image was assigned to a device and only the number of the device was recorded at the demonstration step. The colour of the preferred male in the demonstration was recorded at the end of the experiment.

Replicates met our quality criteria if a copulation occurred during the mate-choice test after both males courted (*i.e.,* wing flapped) the observer female, as this was the only situation when observer females were in a real situation of choice. Other situations were discarded. We tested in total 3407 observer females and discarded 2215 replicates where only one male courted the female (1671 cases) or no copulation was observed within the 30-min mate-choice test (544 cases). Replicates, where the observer female copulated with the male of the phenotype preferred during the demonstration (copied), were attributed a mate-copying score of 1, versus 0 in the opposite case. Then a mate-copying index was calculated as the average of the mate-copying scores per treatment.

#### *Experiment 1—Do females copy from photos?*

We used 18 duets of photos containing two couples of the same colour plus a rejected male of the opposite colour as top and front view (Fig. 2a) on a white background. The demonstration phase lasted 30 min to ensure that females had enough time to observe.

#### *Experiment 2—Does the duration of the demonstration affect copying skills?*

We used the same photos as in Experiment 1 but showed them for either 5 min, 15 min or 30 min. A normal copulation lasts roughly 25 min^27^. Thus, 5 min was much shorter than an average copulation, 15 min slightly shorter and 30 min slightly longer.

#### *Experiments 3–5—How far can we simplify the stimulus without losing its ability to elicit mate copying?*

Simplifications involved a photo of one copulating pair plus one rejected male either in a top or in a side view on white background per photo for 20 min to observer females. In Experiment 3, we used photos of normal flies (Fig. 2a), images where males had a big colour dot on their back (hereafter called dots, Fig. 2b) and images where the whole body of all flies were painted in green and pink respectively (hereafter called painted, Fig. 2c). In Experiment 4, we modified the dots condition by removing the eyes of males (Fig. 2d), or those of the female (Fig. 2e), or those of all flies (Fig. 2f) or removing the wings of all flies (Fig. 2g). Finally, in Experiment 5, we modified the normal photos by removing the eyes of either males (Fig. 2h) or females (Fig. 2i) or removing the wings of males (Fig. 2j) and female (Fig. 2k).

### Statistical analysis

Data were analysed with the R software version 4.0.2^52^. For each condition, the difference from random choice was analysed with a binomial test. Mate-copying scores were then analysed in generalized linear mixed models (GLMM) with binary logistic regression (package *lme4*^53^). A random block effect was introduced into the models to account for the non-independence of observer flies from the same block. The significance of fixed effects was tested using Wald chi-square tests implemented in the ANOVA function of the car package^54^. Starting models included treatment, normalized air pressure at the time of the demonstration, the photo ID and wherever possible the experimenter as fixed effects, as well as interactions between these effects. We included air pressure in the model because a previous study^20^ found that air pressure can have an effect on mate copying. Although this effect was not found in other studies with larger data sets (e.g. ^22^. we accounted for this confounding effect. However, removing it from the model does not change the results in general. We did not include temperature, humidity, date, time etc. because we controlled for them in our experimental room and never found a significant effect of any of these factors. We used a backward selection approach using *P* values, first removing one by one the highest order interaction that was farthest from significant. For pairwise post-hoc tests we used a Fisher test and accounted for multiple testing with Holm’s correction.

## Data Availability

The datasets generated during the current study are available in the Dryad repository, https://doi.org/10.5061/dryad.4qrfj6qdv. https://datadryad.org/stash/share/8EBl4-bROo_gyPJaD_GuuGVxhesHZWbfmxOrInXbByk.
